# Supplementary material for: Identifying optimal first-line immune checkpoint inhibitors based regiments for advanced non-small cell lung cancer without oncogenic driver mutations: A systematic review and network meta-analysis
Source: PLoS One. 2023 Apr 18;18(4):e0283719. doi: 10.1371/journal.pone.0283719 (PMC10112813; doi:10.1371/journal.pone.0283719)
Supplement: S1 Checklist — (DOCX) [file pone.0283719.s001.docx]

| **Section and Topic** | **Item #** | **Checklist item** | **Location where item is reported** |
| --- | --- | --- | --- |
| **TITLE** | | |  |
| Title | 1 | **Identifying optimal first-line immune checkpoint inhibitors based regiments for advanced non-small cell lung cancer without oncogenic driver mutations: a systematic review and network meta-analysis** |  |
| **ABSTRACT** | | |  |
| Abstract | 2 | **Background:** Immune checkpoint inhibitors (ICIs) have changed the treatment pattern of advanced and metastatic NSCLC. A series of ICI based therapies have emerged in the first-line treatment field, but the comparative efficacy was unclear.  **Method:** We searched multiple databases and abstracts of major conference proceedings up to Apri1, 2022 for phase III randomised trials of advanced driver-gene wild type NSCLC patients receiving first-line therapy. Outcomes analyzed included progression free survival (PFS), overall survival (OS), and et al.  **Results:** Thirty-two double-blind RCTs were included, involving 18,656 patients assigned to 22 ICI-based first-line regimens. A series of ICI regiments (including ICI plus chemotherapy), ICI monotherapy, doublet ICIs, doublet ICIs plus chemotherapy) emerged, and showed significant PFS and OS benefit than chemotherapy and chemotherapy + bevacizumab (BEV) for advanced wild-type NSCLC. In comprehensive terms of PFS, chemoimmunotherapy (CIT) were significantly more effective than ICI monotherapy and doublet ICIs. In terms of OS for patients with non-squamous NSCLC, pembrolizumab containing CIT was associated with a median rank of the best regimens, and followed by Atezolizumab+BEV based CIT; while for OS in patients with squamous NSCLC, Cemiplimab and sintilimab based CIT were the most effective regimens. For more than 2 years follow-up, the atezolizumab, pembrolizumab, nivolumab and durvalumab containing ICI therapy all provide a durable long-term OS benefit over chemotherapy and BEV + chemotherapy.  **Conclusions:** The findings of the present NMA represent the most comprehensive evidence, which might suggest or provide basis for first-line ICI therapy decision for advanced NSCLC patients without oncogenic driver mutations. |  |
| **INTRODUCTION** | | |  |
| Rationale | 3 | There remains a lot of controversy about the best therapeutic model (i.e. immunotherapy as single agent or in combination, and how to choose optimal combination therapy), and the differences in treatment-related adverse events between individual regiments. With the marketing of new ICI regiments, the most recent clinical trials findings, and long-term outcomes updated recently , we conducted an updated network meta-analysis to compare the treatment efficacy of different first-lines regimens for advanced NSCLC patients without oncogenic driver mutations. |  |
| Objectives | 4 | To perform a network meta-analysis (NMA) to compare the treatment efficacy of first-line therapies for advanced NSCLC patients without oncogenic  drivers. |  |
| **METHODS** | | |  |
| Eligibility criteria | 5 | The inclusion criteria were as follows: studies that analyzing the efficacy and safety of ICIs (ICI monotherapy or double ICIs) alone or combined with chemotherapy and or antiangiogenetic drugs, as first-line treatments for advanced NSCLC; and reports contained patients’ data, complete protocol, and at least one of key clinical outcomes, such as progression-free survival (PFS), overall survival (OS), and objective response rate (ORR), as well as the incidence of treatment-related adverse events (TRAEs). Phase III trials that analyzing the efficacy of chemotherapy in combination with BEV or placebo as first-line therapy for nonsquamous NSCLC were also included for comparison and to form a connected network.  The following studies were excluded from this review: those involving previously treated patients with advanced NSCLC; studies involving operable NSCLC who were treated with ICI therapy before surgery or after surgery; studies not reported key clinical outcomes (PFS, OS, ORR, the incidence of TRAEs); and studies lacking valid data for evaluating the safety and efficacy of ICI first-line treatment. |  |
| Information sources | 6 | We searched PubMed, the Cochrane Library, Embase, the World Health Organization (WHO) International Clinical Trials Registry Platform (ICTRP), including conference proceedings of the World Conference on Lung Cancer (WCLC), the American Society of Clinical Oncology (ASCO), the European Society of Medical Oncology (EMSO), and the Chinese Society of Clinical Oncology (CSCO) Academic Annual Conference. |  |
| Search strategy | 7 | We searched these databases from inception until Apri1, 2022, with no language restrictions. |  |
| Selection process | 8 | The following Medical Subject Headings were used to search: non-small cell lung cancer (including non-squamous lung cancer, squamous lung cancer), and immunotherapy (including all currently known ICIs: pembrolizumab, atezolizumab, ipilimumab, nivolumab, avelumab, sintilimab, durvalumab, tremelimumab, tislelizumab, toripalimab, sugemalimab, camrelizumab, and et al). Reference lists of relevant studies were also screened. |  |
| Data collection process | 9 | Two investigators (YL and WF) independently examined the titles and abstracts of retrieved articles to assess the eligibility. The full articles were evaluated if a decision could not be made based on the titles and abstracts. Data were extracted by the same two reviewers (YL and WF) using a predefined spread sheet. The extracted data included: trial name, year of publication, study design, number of participants, median/mean age, percent males, histology type, disease stage, PD-L1 expression level, median duration of follow-up, and outcome data. Outcome data including PFS, OS, and ORR were extracted; TRAEs associated with each intervention were also extracted in this network meta-analysis. |  |
| Data items | 10a | Data were extracted by the same two reviewers (YL and WF) using a predefined spread sheet. The extracted data included: trial name, year of publication, study design, number of participants, median/mean age, percent males, histology type, disease stage, PD-L1 expression level, median duration of follow-up, and outcome data. Outcome data including PFS, OS, and ORR were extracted; TRAEs associated with each intervention were also extracted in this network meta-analysis. |  |
|  | 10b | The full texts screen was performed, when a decision could not determine based on the titles and abstracts. Two pairs of investigators (YL, WF, SWM ans SLL) extracted data from the studies or from supplementary materials, and evaluated the risk of bias. Disagreements were resolved through discussion. |  |
| Study risk of bias assessment | 11 | We use the data of PFS, and ORR assessed by blinded independent central review (BICR) or independent radiographic review committee (IRRC), if available. Otherwise, investigator-assessed PFS, and ORR were used. |  |
| Effect measures | 12 | Hazard ratio (HRs) and 95% confidence intervals (CIs) for PFS and OS were extracted, percentage for long-term PFS and OS rate were also extracted. Likewise, dichotomous ORR data, grade ≥3 AEs and immune-mediated were clustered. |  |
| Synthesis methods | 13a | We performed fixed-effects network meta-analyses (NMA) in our study.  According to trial characteristics, heterogeneity analysis was performed on all eligible studies. The NMA meets the technical requirement that each treatment should be represented by at least one clinical study to create a viable comparison network. Under the assumption of consistency, the NMA model associated data from the individual studies with basic parameters that reflect the (pooled) relative treatment effect of each intervention compared with the reference therapy. Based on these parameters, the relative treatment effects of each contrast treatment in the network were obtained. Under the Bayesian framework, we could calculate the probability of being the best treatment out of all treatments included in the connected network. It is also possible to calculate which of all the interventions included in the connected network are the best and which are the next best. The ‘rank probability’ function was performed to calculate rank-probabilities, for each MCMC iteration, the treatments are ranked according to their effect relative to baseline. A frequency table was constructed according to these rankings and normalized by the number of iterations to give the rank probabilities. |  |
|  | 13b | Additional subgroups analyses were performed according to histology (i.e., squamous/non-squamous) and levels of PD-L1 expression (i.e., PD-L1-high: PD-L1 ≥ 50%; PD-L1-negative: PD-L1 < 1%; PD-L1-intermediate: 50%>PD-L1 ≥ 1%). Statistical analysis was carried out using the ‘gemtc’ version 1.0-1 of R-4.1.1 software. |  |
| Reporting bias assessment | 14 | Three histology-based evidence networks were constructed in our analysis: a mixed-histology network, a squamous NSCLC network and a non-squamous NSCLC network. The results of the NMA for PFS and OS were presented with estimates of the treatment effects of each intervention with respect to the reference therapy, chemotherapy. The posterior distributions of relative treatment effects were performed by the median and 95% CIs, which were made up of the 2.5th and 97.5th percentiles of the posterior distributions. If the 95% CIs were completely below or above 1, the respective estimated ratios were considered significant. Additionally, we used SUCRA to perform the rank of all treatments, the closer the SUCRA value is to 1, the better the treatment effect is. |  |
| Certainty assessment | 15 | - |  |
| **RESULTS** | | |  |
| Study selection | 16a | A total of 2,375 citations were identified by the comprehensive search strategy (Figure 1). After excluding duplicate citations, 1765 citations underwent title/abstract screening and 65 studies were retrieved for full text screen. Of the 69 full-text articles screened, 37 studies not meeting the inclusion criteria were excluded. |  |
|  | 16b | Overall, 32 first-line phase III RCTs (comprising 18,656 participants) done between 2016 and 2022 were included in the analysis. Study characteristics of included trials are listed in Tables 1. The mean sample size was range from 1274 to 305 participants. |  |
| Study characteristics | 17 | All 32 trials were randomized controlled phase 3 trials with an open-label (17 studies) or a double-blind (15 studies) trial design . Most of the trials had low risk of bias on the basis of the Cochrane’s tool for randomized trials, and the majority of participants with ECOG performance scores (PS) of 0 or 1. All the RCTs included was completed in the recent 5 years. The majority of included trials (22 of 32 studies) were global, multicentre designed trials , and 10 trials were multicentre trials conducted in China and reported (or were assumed) to study 100% Chinese patients. |  |
| Risk of bias in studies | 18 | We use the data of PFS, and ORR assessed by blinded independent central review (BICR) or independent radiographic review committee (IRRC), if available. Otherwise, investigator-assessed PFS, and ORR were used. Hazard ratio (HRs) and 95% confidence intervals (CIs) for PFS and OS were extracted, percentage for long-term PFS and OS rate were also extracted. Likewise, dichotomous ORR data, grade ≥3 AEs and immune-mediated were clustered. |  |
| Results of individual studies | 19 | A series of ICI regiments (including ICI plus chemotherapy), ICI monotherapy, doublet ICIs, doublet ICIs plus chemotherapy) emerged, and showed significant PFS and OS benefit than chemotherapy and chemotherapy + bevacizumab (BEV) for advanced wild-type NSCLC. In comprehensive terms of PFS, chemoimmunotherapy (CIT) were significantly more effective than ICI monotherapy and doublet ICIs. In terms of OS for patients with non-squamous NSCLC, pembrolizumab containing CIT was associated with a median rank of the best regimens, and followed by Atezolizumab+BEV based CIT; while for OS in patients with squamous NSCLC, Cemiplimab and sintilimab based CIT were the most effective regimens. For more than 2 years follow-up, the atezolizumab, pembrolizumab, nivolumab and durvalumab containing ICI therapy all provide a durable long-term OS benefit over chemotherapy and BEV + chemotherapy. |  |
| Results of syntheses | 20a | (1) A series of ICI regiments (including ICI plus chemotherapy, doublet ICIs, doublet ICIs plus chemotherapy) emerged, and showed significant PFS and OS benefit than chemotherapy and chemotherapy +BEV for advanced wild-type NSCLC. |  |
|  | 20b | (2) In comprehensive terms of PFS, CIT were significantly more effective than ICI monotherapy and doublet ICIs, penpulimab based CIT and ABCP showed numerical superiority over other ICI regimens for PFS. |  |
|  | 20c | (3)In terms of OS for patients with non-squamous NSCLC, pembrolizumab containing CIT ranked to be the best treatment regimen, and followed by sintilimab based CIT; while for OS in patients with squamous NSCLC, camrelizumab and sintilimab based CIT were the most effective regimens. Cemiplimab monotherapy showed numerical superiority over other CIT regimens for OS in patients with high PD-L1 TPS expression. |  |
|  | 20d | (4) No statistically significant benefit for OS or PFS was observed for doublet ICIs based therapies comparing with single ICI based therapies.. (5) For >24 months follow-up, the atezolizumab, pembrolizumab, nivolumab and durvalumab containing ICI therapy (including monotherapy, CIT, doublet ICIs, doublet ICIs plus chemotherapy) all provide a durable, clinically meaningful long-term OS benefit over chemotherapy and BEV+ chemotherapy. |  |
| Reporting biases | 21 | Present assessments of risk of bias due to missing results (arising from reporting biases) for each synthesis assessed. |  |
| Certainty of evidence | 22 | Present assessments of certainty (or confidence) in the body of evidence for each outcome assessed. |  |
| **DISCUSSION** | | |  |
| Discussion | 23a | This NMA is based on 32 first-line RCTs for advanced NSCLC, which involving 18,656 patients randomized to 22 ICI-based regimens with 13 different ICI agents, and including recent published data of long-term follow-up of immunotherapy trials. The present analysis is substantially more comprehensive than previous meta-analysis for first-line ICI therapy for advanced NSCLC [2,3,22,23]. The much larger evidence base, obtained through exhaustive search for published article and abstracts from recent major conference proceedings of the ASCO, EMSO, and WCLC. The main findings of this study including: (1) A series of ICI regiments (including ICI plus chemotherapy, doublet ICIs, doublet ICIs plus chemotherapy) emerged, and showed significant PFS and OS benefit than chemotherapy and chemotherapy +BEV for advanced wild-type NSCLC. (2) In comprehensive terms of PFS, CIT were significantly more effective than ICI monotherapy and doublet ICIs, penpulimab based CIT and ABCP showed numerical superiority over other ICI regimens for PFS. (3) In terms of OS for patients with non-squamous NSCLC, pembrolizumab containing CIT ranked to be the best treatment regimen, and followed by sintilimab based CIT; while for OS in patients with squamous NSCLC, camrelizumab and sintilimab based CIT were the most effective regimens. Cemiplimab monotherapy showed numerical superiority over other CIT regimens for OS in patients with high PD-L1 TPS expression. (4) No statistically significant benefit for OS or PFS was observed for doublet ICIs based therapies comparing with single ICI based therapies. (5) For >24 months follow-up, the atezolizumab, pembrolizumab, nivolumab and durvalumab containing ICI therapy (including monotherapy, CIT, doublet ICIs, doublet ICIs plus chemotherapy) all provide a durable, clinically meaningful long-term OS benefit over chemotherapy and BEV+ chemotherapy. |  |
|  | 23b | Our review has some limitations. First, most of the recent published data with short duration of follow-up, the number of OS events was not mature and the long-term PFS and OS rates were not available. The short follow-up prevented the full assessment of specific ICI regiment. Second, the majority studies included implement the 22C3 pharmDx assay to test the PD-L1 TPS, which is defined based on the proportion of tumor cells in membranous PD-L1 staining.38 PD-L1 positivity refer to a tumour proportion score of 1% or higher. Whereas, IMpower studies utilize SP142 assaying to test PD-L1 level [15,17,40]. Different assay method and reference value might lead to misclassification, affecting outcome assessment. Third, the different disease stage of study cohort may also be a confounding factor. Keynote, IMpower, and CheckMate studies entrolled stage IV NSCLC, whereas others included patients with stage IIIB or IIIC disease, meaning that the comparison need to be interpreted with great caution. Fouth, we lacked data from head-to-head comparisons of these ICIs regiments, many conclusions are reliant on indirect comparisons, and thus the results need to be taken with caution. Last, non-Asian patients were not recruited in the ORIENT, RATIONALE, Camel and CHOICE studies, and the results limited to Chinese subpopulation and therefore should be interpreted with caution. |  |
|  | 23c | The findings from comparisons among ICI regiments should be interpreted by the potential limitations of the study design, and the heterogenous patient populations |  |
|  | 23d | The findings from this network meta-analysis represent the most comprehensive currently available evidence base to guide the initial choice about first-line immunotherapies for advanced NSCLC patients without known EGFR mutations or ALK translocations.  The results of the present NMA may assist the initial choice about first-line immunotherapies for advanced NSCLC patients. |  |
| **OTHER INFORMATION** | | |  |
| Registration and protocol | 24a | This study is registered with PROSPERO, number CRD42021291015, and is reported according to the Preferred Reporting Items for Systematic Reviews and Meta-Analyses (PRISMA) extension statement for network meta-analysis. |  |
|  | 24b | a protocol was not prepared. |  |
| Support | 25 | This work was supported by Natural Science Foundation of Shandong Province (No. ZR2021MH006). |  |
| Competing interests | 26 | The authors declare that they have no conflict of interest. |  |
| Availability of data, code and other materials | 27 | All data included in this study are available upon request by contact with the corresponding author. |  |
