# Supplementary material for: Identifying optimal first-line immune checkpoint inhibitors based regiments for advanced non-small cell lung cancer without oncogenic driver mutations: A systematic review and network meta-analysis
Source: PLoS One. 2023 Apr 18;18(4):e0283719. doi: 10.1371/journal.pone.0283719 (PMC10112813; doi:10.1371/journal.pone.0283719)
Supplement: S2 Table — (DOC) [file pone.0283719.s003.doc]

| **PFS** | | | | | | | | | | | | | | | | | | | | |
| --- | --- | --- | --- | --- | --- | --- | --- | --- | --- | --- | --- | --- | --- | --- | --- | --- | --- | --- | --- | --- |
| **OS** | PENP+ChT | 0.90  (0.63, 1.3) | 0.88  (0.61, 1.3) | 0.79  (0.55, 1.1) | 0.77  (0.55, 1.1) | 0.74  (0.49, 1.1) | 0.71  (0.48, 1.1) | 0.71  (0.47, 1.1) | **0.69**  **(0.46, 1.0)** | **0.61**  **(0.44, 0.85)** | **0.56**  **(0.39, 0.79)** | **0.55**  **(0.40, 0.76)** | **0.54**  **(0.38, 0.77)** | **0.48**  **(0.34, 0.68)** | **0.46**  **(0.28, 0.76)** | **0.46**  **(0.33, 0.65)** | **0.38**  **(0.23, 0.62)** | **0.37**  **(0.27, 0.52)** | **0.35**  **(0.24, 0.51)** | **0.40**  **(0.29, 0.55)** |
|  | — | ATEZ+BCP | 0.97  (0.75, 1.3) | 0.87  (0.67, 1.1) | 0.85  (0.67, 1.1) | 0.82  (0.59, 1.1) | 0.79  (0.59, 1.1) | 0.79  (0.56, 1.1) | **0.76**  **(0.56, 1.0)** | **0.68**  **(0.55, 0.84)** | **0.61**  **(0.47, 0.80)** | **0.61**  **(0.50, 0.75)** | **0.60**  **(0.46, 0.78)** | **0.53**  **(0.42, 0.68)** | **0.51**  **(0.33, 0.78)** | **0.51**  **(0.40, 0.65)** | **0.42**  **(0.28, 0.64)** | **0.41**  **(0.33, 0.52)** | **0.38**  **(0.29, 0.52)** | **0.44**  **(0.37, 0.53)** |
|  | — | 1.1  (0.76, 1.7) | CAMR+ChT | 0.89  (0.69, 1.2) | 0.87  (0.69, 1.1) | 0.84  (0.61, 1.2) | 0.81  (0.60, 1.1) | 0.81  (0.58, 1.1) | 0.78  (0.58, 1.1) | **0.70**  **(0.56, 0.86)** | **0.63**  **(0.49, 0.81)** | **0.63**  **(0.51, 0.77)** | **0.61**  **(0.47, 0.79)** | **0.55**  **(0.43, 0.69)** | **0.52**  **(0.34, 0.80)** | **0.52**  **(0.41, 0.66)** | **0.43**  **(0.29, 0.66)** | **0.42**  **(0.34, 0.53)** | **0.40**  **(0.29, 0.53)** | **0.45**  **(0.38, 0.55)** |
|  | — | 1.2  (0.82, 1.8) | 1.1  (0.63, 1.8) | SINT+ChT | 0.97  (0.77, 1.2) | 0.94  (0.68, 1.3) | 0.91  (0.67, 1.2) | 0.91  (0.65, 1.3) | 0.87  (0.64, 1.2) | **0.78**  **(0.63, 0.97)** | **0.71**  **(0.54, 0.92)** | **0.70**  **(0.57, 0.86)** | **0.69**  **(0.53, 0.89)** | **0.61**  **(0.48, 0.78)** | **0.58**  **(0.38, 0.90)** | **0.58**  **(0.46, 0.74)** | **0.48**  **(0.32, 0.74)** | **0.47**  **(0.38, 0.60)** | **0.44**  **(0.33, 0.60)** | **0.51**  **(0.42, 0.62)** |
|  | — | 1.2  (0.96, 1.5) | 1.0  (0.70, 1.6) | 0.99  (0.67, 1.5) | PEMB+ChT | 0.97  (0.71, 1.3) | 0.93  (0.71, 1.2) | 0.93  (0.68, 1.3) | 0.90  (0.68, 1.2) | **0.80**  **(0.67, 0.95)** | **0.72**  **(0.58, 0.91)** | **0.72**  **(0.61, 0.85)** | **0.70**  **(0.56, 0.88)** | **0.63**  **(0.51, 0.77)** | **0.60**  **(0.40, 0.91)** | **0.60**  **(0.49, 0.73)** | **0.50**  **(0.33, 0.74)** | **0.49**  **(0.40, 0.59)** | **0.45**  **(0.35, 0.59)** | **0.52**  **(0.45, 0.60)** |
|  | — | 1.1  (0.72, 1.7) | 0.97  (0.56, 1.7) | 0.92  (0.54, 1.6) | 0.92  (0.60, 1.4) | SUGE+ChT | 0.96  (0.68, 1.4) | 0.96  (0.66, 1.4) | 0.93  (0.65, 1.3) | 0.83  (0.62, 1.1) | **0.75**  **(0.54, 1.0)** | **0.75**  **(0.56, 0.99)** | **0.73**  **(0.53, 1.0)** | **0.65**  **(0.48, 0.88)** | **0.62**  **(0.39, 1.0)** | **0.62**  **(0.46, 0.84)** | **0.51**  **(0.32, 0.82)** | **0.50**  **(0.38, 0.68)** | **0.47**  **(0.33, 0.67)** | **0.54**  **(0.41, 0.70)** |
|  | — | 1.0  (0.75, 1.4) | 0.90  (0.56, 1.4) | 0.85  (0.54, 1.3) | 0.86  (0.62, 1.2) | 0.93  (0.57, 1.5) | CEMI+ChT | 1.0  (0.70, 1.4) | 0.96  (0.69, 1.3) | 0.86  (0.67, 1.1) | **0.78**  **(0.58, 1.0)** | **0.77**  **(0.60, 0.99)** | **0.76**  **(0.56, 1.0)** | **0.67**  **(0.51, 0.89)** | **0.64**  **(0.41, 1.0)** | **0.64**  **(0.49, 0.85)** | **0.53**  **(0.34, 0.83)** | **0.52**  **(0.40, 0.68)** | **0.49**  **(0.35, 0.68)** | **0.56**  **(0.44, 0.71)** |
|  | — | — | — | — | — | — | — | TORI+ChT | 0.96  (0.67, 1.4) | 0.86  (0.64, 1.2) | 0.78  (0.56, 1.1) | **0.77**  **(0.58, 1.0)** | 0.76  (0.54, 1.1) | **0.67**  **(0.49, 0.92)** | **0.64**  **(0.40, 1.0)** | **0.64**  **(0.47, 0.88)** | **0.53**  **(0.33, 0.85)** | **0.52**  **(0.39, 0.71)** | **0.49**  **(0.34, 0.70)** | **0.56**  **(0.42, 0.74)** |
|  | — | — | — | — | — | — | — | — | TISL+ChT | 0.89  (0.69, 1.2) | 0.81  (0.60, 1.1) | **0.80**  **(0.62, 1.0)** | 0.79  (0.58, 1.1) | **0.70**  **(0.53, 0.93)** | 0.67  (0.42, 1.1) | **0.67**  **(0.50, 0.89)** | **0.55**  **(0.35, 0.87)** | **0.54**  **(0.41, 0.71)** | **0.51**  **(0.36, 0.71)** | **0.58**  **(0.46, 0.74)** |
|  | — | **0.90**  **(0.79, 1.0)** | 0.79  (0.53, 1.2) | **0.73**  **(0.52, 1.0)** | **0.75**  **(0.62, 0.91)** | 0.81  (0.54, 1.2) | 0.88  (0.65, 1.2) | — | — | ATEZ+ChT | 0.91  (0.74, 1.1) | **0.90**  **(0.79, 1.0)** | 0.88  (0.72, 1.1) | **0.79**  **(0.66, 0.94)** | 0.75  (0.50, 1.1) | **0.75**  **(0.62, 0.90)** | **0.62**  **(0.42, 0.92)** | **0.61**  **(0.52, 0.72)** | **0.57**  **(0.44, 0.73)** | **0.65**  **(0.59, 0.72)** |
|  | — | 0.95  (0.75, 1.2) | 0.83  (0.55, 1.1) | 0.79  (0.53, 1.2) | **0.79**  **(0.63, 1.0)** | 0.86  (0.56, 1.3) | 0.92  (0.6, 1.3) | — | — | 1.1  (0.85, 1.3) | DURV+  TREM+ChT | 0.99  (0.81, 1.2) | **0.97**  **(0.94, 1.0)** | 0.87  (0.69, 1.1) | 0.83  (0.54, 1.3) | **0.83**  **(0.66, 1.0)** | **0.69**  **(0.45, 1.0)** | **0.67**  **(0.54, 0.84)** | **0.63**  **(0.47, 0.84)** | **0.72**  **(0.60, 0.86)** |
|  | — | 0.86  (0.68, 1.1) | 0.76  (0.49, 1.2) | 0.71  (0.48, 1.1) | **0.72**  **(0.56, 0.92)** | 0.78  (0.50, 1.2) | 0.84  (0.60, 1.2) | — | — | 0.96  (0.77, 1.2) | 0.91  (0.70, 1.2) | ATEZ | 0.98  (0.80, 1.2) | **0.87**  **(0.74, 1.0)** | 0.83  (0.56, 1.2) | **0.83**  **(0.70, 0.99)** | **0.69**  **(0.47, 1.0)** | **0.68**  **(0.58, 0.79)** | **0.63**  **(0.49, 0.81)** | **0.72**  **(0.67, 0.79)** |
|  | — | 0.85  (0.68, 1.1) | 0.75  (0.49, 1.1) | **0.70**  **(0.47, 1.0)** | **0.71**  **(0.56, 0.90)** | 0.77  (0.50, 1.2) | 0.83  (0.60, 1.2) | — | — | 0.94  (0.76, 1.2) | **0.90**  **(0.87, 0.92)** | 0.98  (0.76, 1.3) | DURV+ChT | 0.89  (0.71, 1.1) | 0.85  (0.55, 1.3) | 0.85  (0.67, 1.1) | 0.71  (0.47, 1.1) | **0.69**  **(0.55, 0.86)** | **0.64**  **(0.48, 0.86)** | **0.74**  **(0.62, 0.88)** |
|  | — | 0.97  (0.80, 1.2) | 0.85  (0.57, 1.3) | 0.80  (0.55, 1.2) | **0.81**  **(0.66, 0.99)** | 0.88  (0.58, 1.3) | 0.94  (0.69, 1.3) | — | — | 1.1  (0.91, 1.3) | 1.0  (0.82, 1.3) | 1.1  (0.89, 1.4) | 1.1  (0.92, 1.4) | NIVO+IPIL | 0.95  (0.63, 1.4) | 0.95  (0.78, 1.2) | 0.79  (0.53, 1.2) | **0.78**  **(0.64, 0.94)** | **0.72**  **(0.55, 0.95)** | **0.83**  **(0.72, 0.96)** |
|  | — | 0.96  (0.69, 1.3) | 0.84  (0.52, 1.4) | 0.80  (0.50, 1.3) | 0.80  (0.57, 1.1) | 0.87  (0.53, 1.4) | 0.93  (0.62, 1.4) | — | — | 1.1  (0.78, 1.5) | 1.0  (0.72, 1.4) | 1.1  (0.78, 1.6) | 1.1  (0.80, 1.6) | 0.99  (0.71, 1.4) | DURV | 1.0  (0.66, 1.5) | 0.83  (0.48, 1.4) | 0.81  (0.54, 1.2) | 0.76  (0.48, 1.2) | 0.87  (0.59, 1.3) |
|  | — | **0.80**  **(0.64, 0.99)** | 0.70  (0.46, 1.1) | **0.66**  **(0.45, 0.98)** | **0.67**  **(0.53, 0.84)** | 0.73  (0.47, 1.1) | 0.78  (0.56, 1.1) | — | — | 0.89  (0.73, 1.1) | 0.85  (0.67, 1.1) | 0.93  (0.72, 1.2) | 0.95  (0.75, 1.2) | **0.83**  **(0.67, 1.0)** | 0.83  (0.59, 1.2) | IPIL+ChT | 0.83  (0.55, 1.2) | **0.81**  **(0.67, 0.99)** | **0.76**  **(0.57, 1.0)** | **0.87**  **(0.75, 1.0)** |
|  | — | 0.85  (0.60, 1.2) | 0.75  (0.46, 1.2) | 0.71  (0.44, 1.2) | **0.72**  **(0.50, 1.0)** | 0.78  (0.47, 1.3) | 0.84  (0.54, 1.3) | — | — | 0.95  (0.68, 1.3) | 0.90  (0.63, 1.3) | 1.0  (0.68, 1.4) | 1.0  (0.70, 1.5) | 0.88  (0.62, 1.3) | 0.89  (0.57, 1.4) | 1.1  (0.74, 1.5) | DURV+  TREM | 0.98  (0.66, 1.5) | 0.91  (0.59, 1.4) | 1.1  (0.72, 1.5) |
|  | — | 0.90  (0.74, 1.1) | 0.79  (0.53, 1.2) | 0.75  (0.51, 1.1) | **0.75**  **(0.61, 0.93)** | 0.81  (0.54, 1.2) | 0.88  (0.64, 1.2) | — | — | 1.0  (0.84, 1.2) | 0.95  (0.77, 1.2) | 1.0  (0.83, 1.3) | 1.1  (0.85, 1.3) | 0.93  (0.77, 1.1) | 0.94  (0.68, 1.3) | 1.1  (0.91, 1.4) | 1.0  (0.74, 1.5) | PEMB | 0.93  (0.71, 1.2) | 1.1  (0.94, 1.2) |
|  | — | **0.81**  **(0.64, 1.0)** | 0.71  (0.47, 1.1) | **0.67**  **(0.45, 1.0)** | **0.68**  **(0.53, 0.86)** | 0.73  (0.47, 1.1) | 0.79  (0.56, 1.1) | — | — | 0.90  (0.73, 1.1) | 0.85  (0.67, 1.1) | 0.94  (0.72, 1.2) | 0.95  (0.74, 1.2) | **0.83**  **(0.69, 1.0)** | 0.84  (0.59, 1.2) | 1.0  (0.79, 1.3) | 0.94  (0.65, 1.4) | 0.90  (0.72, 1.1) | NIVO | 1.2  (0.91, 1.5) |
|  | — | **0.73**  **(0.63, 0.84)** | **0.64**  **(0.44, 0.94)** | **0.60**  **(0.43, 0.86)** | **0.61**  **(0.52, 0.71)** | **0.66**  **(0.45, 0.98)** | **0.71**  **(0.54, 0.94)** | — | — | **0.81**  **(0.72, 0.91)** | **0.77**  **(0.65, 0.92)** | **0.85**  **(0.70, 1.0)** | **0.86**  **(0.72, 1.0)** | **0.75**  **(0.66, 0.86)** | **0.76**  **(0.56, 1.0)** | 0.91  (0.77, 1.1) | 0.85  (0.61, 1.2) | **0.81**  **(0.71, 0.93)** | 0.90  (0.76, 1.1) | ChT |

**S2 Table. PFS, and OS comparative profiles for overall study cohort according to network meta-analysis (NMA).**

Each cell contains the Hazard-Radio (HR) and 95% credibility intervals for PFS and OS; significant results are emboldened.

Abbreviation: ATEZ, atelizumab; BEV, bevacizumab; CAMR, camrelizumab; CEMI, cemiplimab; ChT, ChT; DURV, durvalumab; IPIL, ipilimumab; NIVO, nivolumab; PEMB, pembrolizumab; PENP, Penpulimab; SINT, sintilimab; SUGE, sugemalimab;TISL, Tislelizumab; TORI, Toripalimab; TREM, tremelimumab.
