# Supplementary material for: Identifying optimal first-line immune checkpoint inhibitors based regiments for advanced non-small cell lung cancer without oncogenic driver mutations: A systematic review and network meta-analysis
Source: PLoS One. 2023 Apr 18;18(4):e0283719. doi: 10.1371/journal.pone.0283719 (PMC10112813; doi:10.1371/journal.pone.0283719)
Supplement: S3 Table — (DOC) [file pone.0283719.s004.doc]

| **Squamous** | | | | | | | | | | | | | | | | |
| --- | --- | --- | --- | --- | --- | --- | --- | --- | --- | --- | --- | --- | --- | --- | --- | --- |
| **Non-squamous** | SUGE+ChT | 0.89  (0.55, 1.4) | 0.94  (0.41, 2.2) | 0.83  (0.49, 1.4) | 0.69  (0.40, 1.2) | 0.64  (0.37, 1.1) | **0.62**  **(0.38, 1.0)** | **0.59**  **(0.34, 1.0)** | **0.59**  **(0.37, 0.95)** | **0.52**  **(0.28, 1.0)** | **0.47**  **(0.30, 0.73)** | **0.40**  **(0.22, 0.72)** | **0.38**  **(0.24, 0.59)** | **0.29**  **(0.15, 0.56)** | — | **0.33**  **(0.22, 0.50)** |
|  | 1.1  (0.72, 1.7) | CAMR+ChT | 1.1  (0.50, 2.2) | 0.92  (0.62, 1.4) | 0.77  (0.51, 1.2) | 0.71  (0.47, 1.1) | **0.70**  **(0.49, 0.99)** | **0.66**  **(0.44, 1.0)** | **0.66**  **(0.48, 0.92)** | **0.59**  **(0.34, 1.0)** | **0.52**  **(0.39, 0.70)** | **0.45**  **(0.27, 0.73)** | **0.43**  **(0.32, 0.56)** | **0.32**  **(0.18, 0.57)** | — | **0.37**  **(0.29, 0.47)** |
|  | 1.2  (0.75, 1.9) | 1.1  (0.71, 1.7) | PEMB | 0.88  (0.40, 1.9) | 0.73  (0.33, 1.6) | 0.67  (0.31, 1.5) | 0.66  (0.31, 1.4) | 0.63  (0.28, 1.4) | 0.63  (0.30, 1.3) | 0.56  (0.23, 1.3) | **0.49**  **(0.24, 1.0)** | **0.42**  **(0.18, 0.97)** | **0.40**  **(0.19, 0.83)** | **0.31**  **(0.13, 0.74)** | — | **0.35**  **(0.17, 0.71)** |
|  | — | — | — | PENP+ChT | 0.83  (0.53, 1.3) | 0.77  (0.48, 1.2) | 0.76  (0.50, 1.1) | 0.71  (0.45, 1.1) | **0.71**  **(0.49, 1.0)** | 0.64  (0.36, 1.1) | **0.56**  **(0.39, 0.80)** | **0.48**  **(0.29, 0.81)** | **0.46**  **(0.33, 0.65)** | **0.35**  **(0.19, 0.64)** | — | **0.40**  **(0.29, 0.54)** |
|  | 1.1  (0.70, 1.7) | 1.0  (0.66, 1.5) | 0.92  (0.58, 1.4) | — | CEMI | 0.92  (0.57, 1.5) | 0.91  (0.59, 1.4) | 0.86  (0.53, 1.4) | 0.86  (0.57, 1.3) | 0.76  (0.42, 1.4) | **0.68**  **(0.46, 0.99)** | **0.58**  **(0.34, 0.99)** | **0.55**  **(0.38, 0.80)** | **0.42**  **(0.23, 0.78)** | — | **0.48**  **(0.34, 0.67)** |
|  | 1.0  (0.64, 1.6) | 0.93  (0.60, 1.4) | 0.85  (0.53, 1.4) | — | 0.93  (0.59, 1.5) | TISL+ChT | 0.98  (0.64, 1.5) | 0.93  (0.57, 1.5) | 0.93  (0.62, 1.4) | 0.83  (0.45, 1.5) | 0.73  (0.50, 1.1) | 0.63  (0.36, 1.1) | **0.60**  **(0.41, 0.87)** | **0.46**  **(0.24, 0.85)** | — | **0.52**  **(0.37, 0.74)** |
|  | 1.4  (0.89, 2.1) | 1.2  (0.83, 1.9) | 1.1  (0.74, 1.8) | — | 1.2  (0.82, 1.9) | 1.3  (0.86, 2.1) | SINT+ChT | 0.95  (0.62, 1.4) | 0.95  (0.68, 1.3) | 0.84  (0.49, 1.5) | **0.75**  **(0.55, 1.0)** | **0.64**  **(0.39, 1.0)** | **0.61**  **(0.45, 0.82)** | **0.46**  **(0.26, 0.83)** | — | **0.53**  **(0.41, 0.68)** |
|  | 1.2  (0.79, 2.0) | 1.1  (0.74, 1.7) | 1.0  (0.66, 1.6) | — | 1.1  (0.73, 1.8) | 1.2  (0.77, 1.9) | 0.91  (0.60, 1.4) | CEMI+ChT | 1.0  (0.67, 1.5) | 0.89  (0.49, 1.6) | 0.79  (0.54, 1.2) | 0.67  (0.39, 1.2) | **0.55**  **(0.38, 0.80)** | **0.49**  **(0.26, 0.91)** | — | **0.56**  **(0.40, 0.79)** |
|  | 1.3  (0.92, 1.9) | 1.2  (0.86, 1.7) | 1.1  (0.76, 1.6) | — | 1.2  (0.85, 1.7) | 1.3  (0.89, 1.9) | 0.97  (0.69, 1.4) | 1.1  (0.74, 1.5) | PEMB+ChT | 0.89  (0.52, 1.5) | **0.79**  **(0.60, 1.0)** | 0.68  (0.42, 1.1) | **0.64**  **(0.49, 0.84)** | **0.49**  **(028, 0.86)** | — | **0.56**  **(0.45, 0.70)** |
|  | 1.2  (0.73, 2.0) | 1.1  (0.69, 1.7) | 1.0  (0.61, 1.6) | — | 1.1  (0.68, 1.8) | 1.2  (0.71, 1.9) | 0.88  (0.55, 1.4) | 0.96  (0.59, 1.6) | 0.91  (0.60, 1.4) | NIVO+IPIL | 0.89  (0.53, 1.5) | 0.76  (0.40, 1.4) | 0.72  (0.43, 1.2) | 0.55  (0.27, 1.1) | — | **0.63**  **(0.39, 1.0)** |
|  | 1.1  (0.75, 1.5) | 0.97  (0.71, 1.3) | 0.89  (0.62, 1.3) | — | 0.97  (0.69, 1.3) | 1.0  (0.73, 1.5) | 0.78  (0.57, 1.1) | 0.85  (0.61, 1.2) | **0.80**  **(0.64, 1.0)** | 0.89  (0.60, 1.3) | ATEZ+ChT | 0.86  (0.54, 1.4) | **0.82**  **(0.65, 1.0)** | 0.62  (0.36, 1.1) | — | **0.71**  **(0.60, 0.84)** |
|  | **0.51**  **(0.34, 0.76)** | **0.47**  **(0.32, 0.67)** | **0.43**  **(0.28, 0.64)** | — | **0.47**  **(0.32, 0.68)** | **0.50**  **(0.33, 0.75)** | **0.37**  **(0.26, 0.54)** | **0.41**  **(0.28, 0.61)** | **0.39**  **(0.29, 0.52)** | **0.43**  **(0.28, 0.66)** | **0.48**  **(0.37, 0.63)** | NIVO | 0.95  (0.61, 1.5) | 0.73  (0.37, 1.4) | — | 0.83  (0.54, 1.3) |
|  | — | — | — | — | — | — | — | — | — | — | — | — | IPIL+ChT | 0.76  (0.44, 1.3) | — | **0.87**  **(0.75, 1.0)** |
|  | 1.0  (0.65, 1.6) | 0.92  (0.61, 1.4) | 0.85  (0.54, 1.3) | — | 0.92  (0.60, 1.4) | 0.99  (0.63, 1.6) | 0.74  (0.49, 1.1) | 0.82  (0.53, 1.3) | 0.77  (0.54, 1.1) | 0.85  (0.52, 1.4) | 0.96  (0.69, 1.3) | **2.0**  **(1.4, 2.9)** | — | ATEZ | — | 1.1  (0.68, 1.9) |
|  | **1.5**  **(1.0, 2.2)** | 1.4  (0.97, 1.9) | 1.2  (0.85, 1.8) | — | 1.4  (0.95, 1.9) | 1.5  (0.99, 2.1) | 1.1  (0.77, 1.5) | 1.2  (0.83, 1.7) | 1.1  (0.87, 1.5) | 1.2  (0.82, 1.9) | **1.4**  **(1.1, 1.8)** | **2.9**  **(2.2, 3.9)** | — | **1.5**  **(1.0, 2.1)** | ATEZ+BCP | — |
|  | **0.66**  **(0.48, 0.91)** | **0.60**  **(0.45, 0.79)** | **0.55**  **(0.39, 0.77)** | — | **0.60**  **(0.44, 0.81)** | **0.65**  **(0.46, 0.90)** | **0.48**  **(0.36, 0.64)** | **0.53**  **(0.39, 0.73)** | **0.50**  **(0.42, 0.59)** | **0.55**  **(0.38, 0.80)** | **0.62**  **(0.55, 0.71)** | **1.3**  **(1.0, 1.6)** | — | **0.65**  **(0.48, 0.88)** | **0.44**  **(0.37, 0.53)** | ChT |

**S3 Table. PFS comparative profiles for squamous and non-squamous cohort according to network meta-analysis (NMA).**

Each cell contains the Hazard-Radio (HR) and 95% credibility intervals for PFS; significant results are emboldened.

Abbreviation: ATEZ, atelizumab; BEV, bevacizumab; CAMR, camrelizumab; CEMI, cemiplimab; ChT, ChT; IPIL, ipilimumab; NIVO, nivolumab; PEMB, pembrolizumab; PENP, Penpulimab; SINT, sintilimab; SUGE, sugemalimab;TISL, Tislelizumab;
