# Supplementary material for: Identifying optimal first-line immune checkpoint inhibitors based regiments for advanced non-small cell lung cancer without oncogenic driver mutations: A systematic review and network meta-analysis
Source: PLoS One. 2023 Apr 18;18(4):e0283719. doi: 10.1371/journal.pone.0283719 (PMC10112813; doi:10.1371/journal.pone.0283719)
Supplement: S4 Table — (DOC) [file pone.0283719.s005.doc]

| **Squamous** | | | | | | | | | |
| --- | --- | --- | --- | --- | --- | --- | --- | --- | --- |
| **Non-squamous** | CEMI | 0.84  (0.42, 1.7) | 0.86  (0.46, 1.6) | 0.84  (0.43, 1.6) | 0.68  (0.40, 1.1) | 0.59  (0.31, 1.1) | **0.55**  **(0.33, 0.90)** | **0.53**  **(0.32, 0.87)** | **0.48**  **(0.30, 0.77)** |
|  | 0.88  (0.44, 1.7) | CAMR+ChT | 1.0  (0.53, 2.0) | 1.0  (0.50, 2.0) | 0.80  (0.46, 1.4) | 0.70  (0.36, 1.4) | 0.65  (0.37, 1.1) | 0.63  (0.36, 1.1) | **0.55**  **(0.34, 0.96)** |
|  | 0.81  (0.47, 1.4) | 0.92  (0.47, 1.8) | CEMI+ChT | 0.98  (0.52, 1.8) | 0.79  (0.50, 1.2) | 0.68  (0.38, 1.2) | **0.64**  **(0.41, 1.0)** | **0.62**  **(0.40, 0.96)** | **0.56**  **(0.37, 0.84)** |
|  | 0.98  (0.51, 1.9) | 1.1  (0.52, 2.4) | 1.2  (0.64, 2.3) | SINT+ChT | 0.80  (0.48, 1.3) | 0.69  (0.37, 1.3) | 0.65  (0.39, 1.1) | **0.63**  **(0.38, 1.0)** | **0.57**  **(0.35, 0.92)** |
|  | 1.3  (0.80, 2.0) | 1.5  (0.79, 2.7) | **1.6**  **(1.0, 2.4)** | 1.2  (0.75, 2.0) | PEMB+ChT | 0.87  (0.55, 1.4) | 0.81  (0.61, 1.1) | **0.78**  **(0.60, 1.0)** | **0.71**  **(0.58, 0.87)** |
|  | **0.55**  **(0.34, 0.88)** | 0.62  (0.34, 1.2) | 0.68  (0.43, 1.1) | **0.56**  **(0.31, 1.0)** | **0.43**  **(0.30, 0.61)** | NIVO | 0.93  (0.59, 1.5) | 0.90  (0.58, 1.4) | 0.82  (0.54, 1.20) |
|  | 0.80  (0.51, 1.2) | 0.91  (0.51, 1.6) | 0.98  (0.65, 1.5) | 0.81  (0.46, 1.4) | **0.63**  **(0.46, 0.85)** | **1.5**  **(1.1, 2.0)** | ATEZ+ChT | 0.97  (0.76, 1.2) | 0.88  (0.73, 1.10) |
|  | — | — | — | — | — | — | — | IPIL+ChT | 0.91  (0.77, 1.10) |
|  | **0.64**  **(0.43, 0.96)** | 0.73  (0.42, 1.30) | 0.79  (0.54, 1.10) | 0.65  (0.38, 1.10) | **0.50**  **(0.40, 0.64)** | 1.2  (0.91, 1.50) | **0.80**  **(0.66, 0.97)** | — | ChT |

**S4 Table. OS comparative profiles for squamous and non-squamous cohort according to network meta-analysis (NMA).**

Each cell contains the Hazard-Radio (HR) and 95% credibility intervals for OS; significant results are emboldened.

Abbreviation: ATEZ, atelizumab; BEV, bevacizumab; CAMR, camrelizumab; CEMI, cemiplimab; ChT, ChT; ; IPIL, ipilimumab; NIVO, nivolumab; PEMB, pembrolizumab; PENP, Penpulimab; SINT, sintilimab;
