# Supplementary figures and images for: Identifying optimal first-line immune checkpoint inhibitors based regiments for advanced non-small cell lung cancer without oncogenic driver mutations: A systematic review and network meta-analysis
Source: PLoS One. 2023 Apr 18;18(4):e0283719. doi: 10.1371/journal.pone.0283719 (PMC10112813; doi:10.1371/journal.pone.0283719)

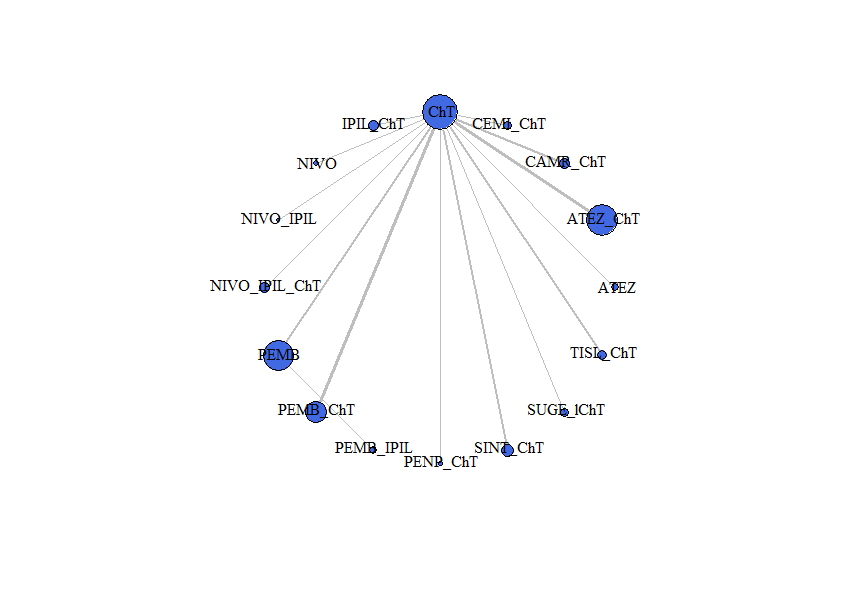

Supplement: S1 Fig — The size of the circle corresponds to to the number of randomly assigned participants (the circle of the chemotherapy group proportional to the number of participants divided by 6). The directly compared interventions are linked with a line, and the thickness of the lines is proportional to the number of trials that assessed the comparison. (TIF) [file pone.0283719.s006.tif]

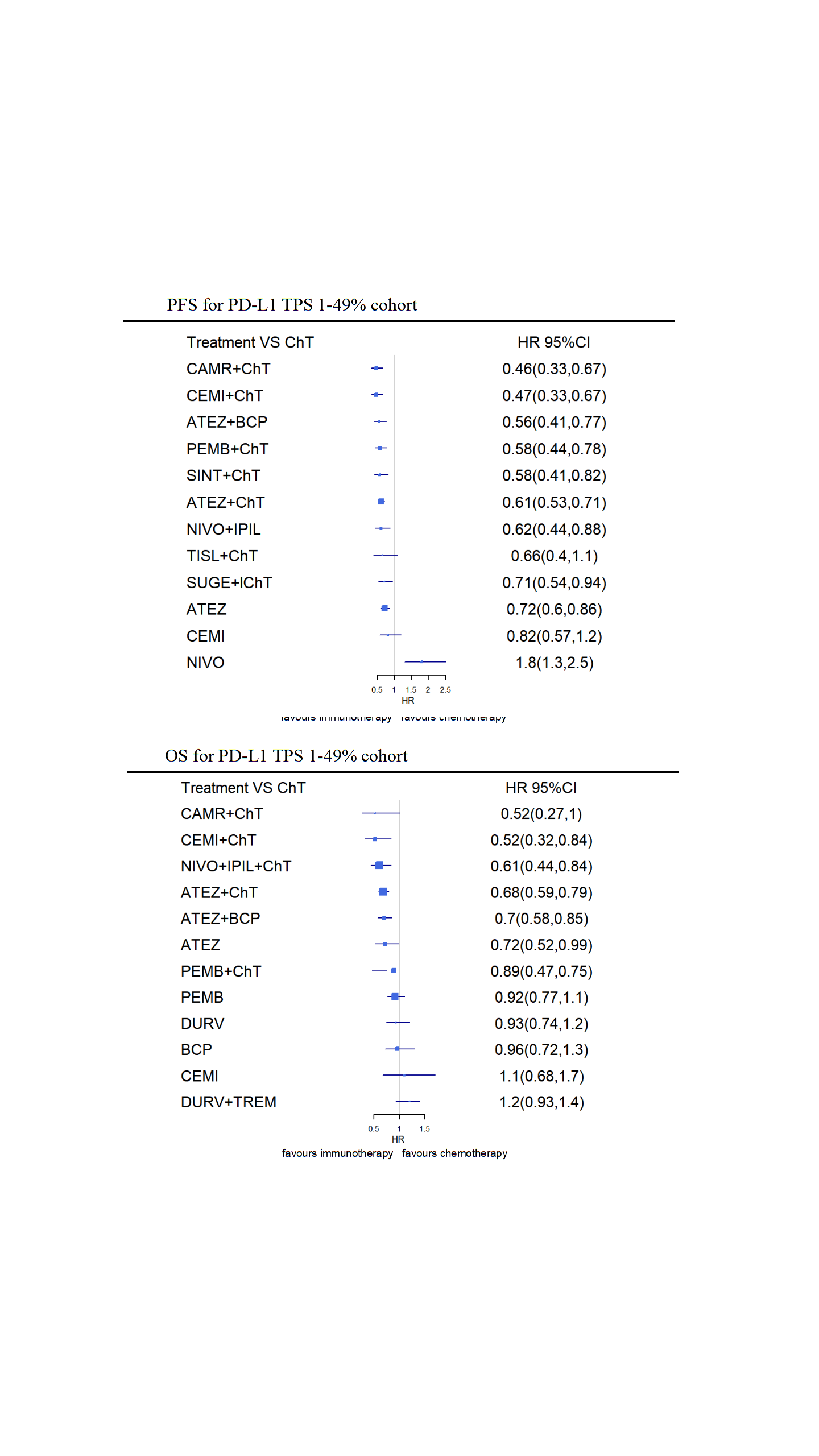

Supplement: S2 Fig — Summary estimates represent Hazard-Radio (HR) and 95% credibility intervals for PFS and OS. Interventions are ranked by Surface Under the Cumulative Ranking curve values. (TIF) [file pone.0283719.s007.tif]

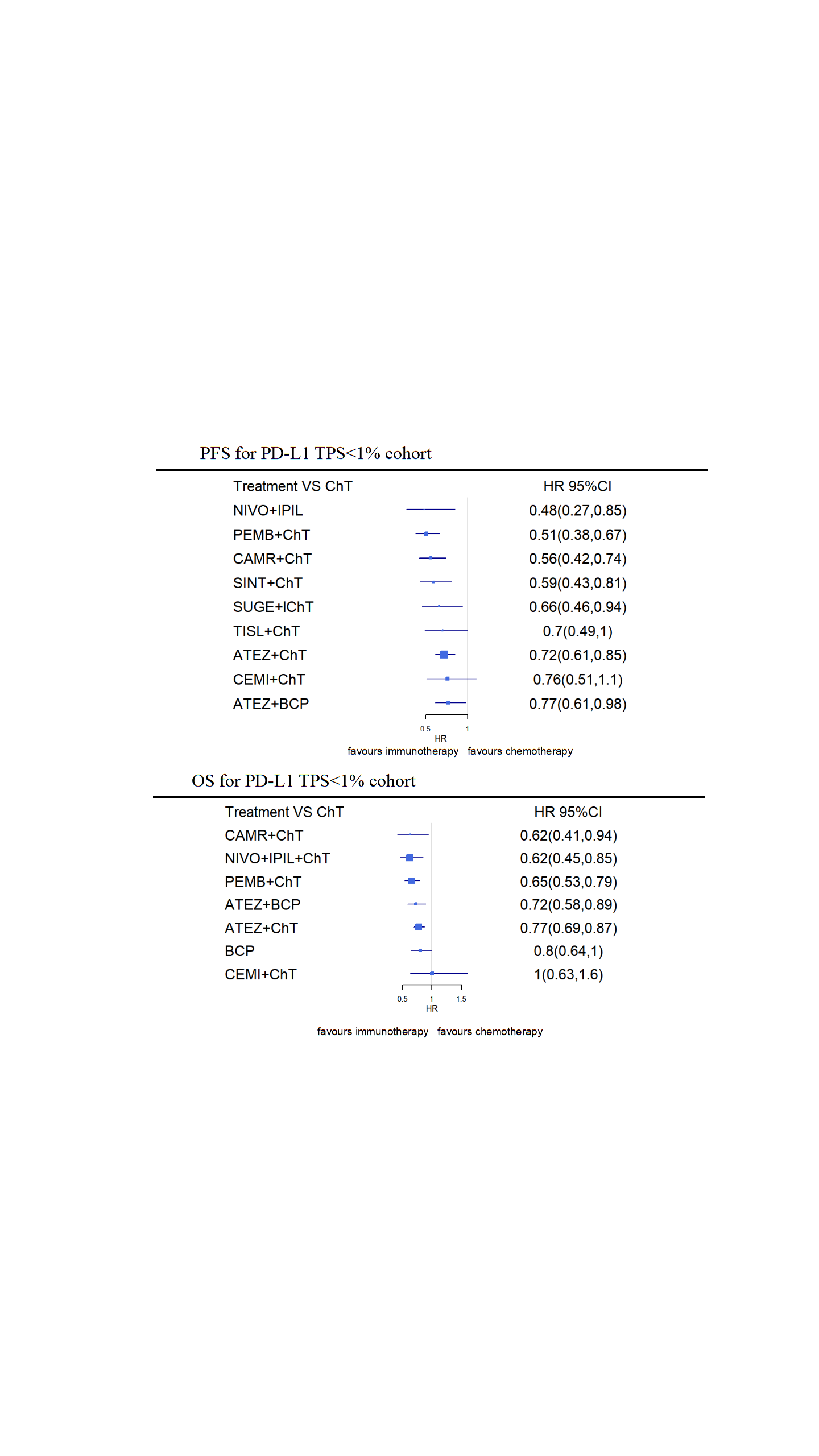

Supplement: S3 Fig — Summary estimates represent Hazard-Radio (HR) and 95% credibility intervals for PFS and OS. Interventions are ranked by Surface Under the Cumulative Ranking curve values. (TIF) [file pone.0283719.s008.tif]

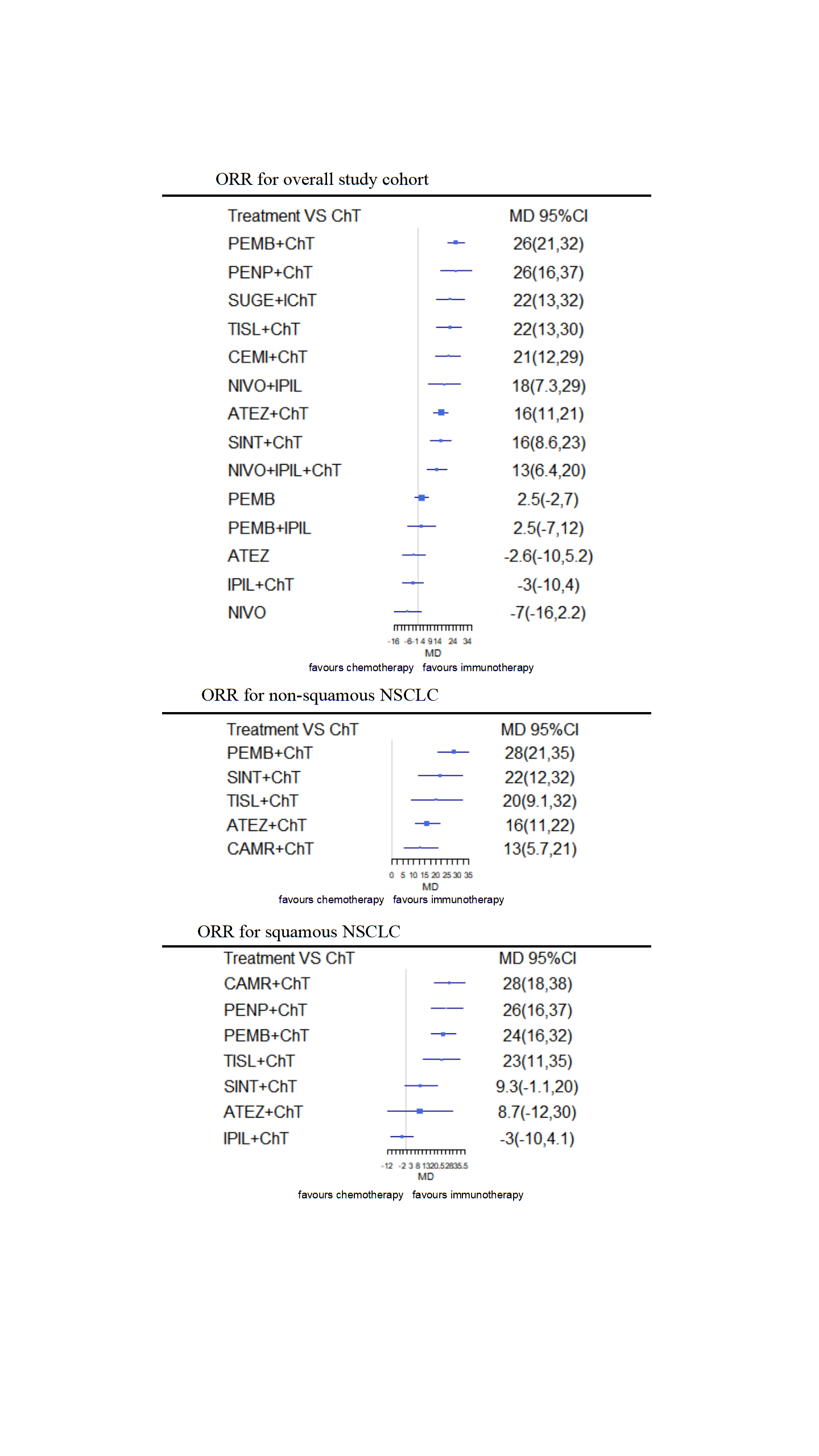

Supplement: S4 Fig — Each cell contains the Mean difference (MD) and 95% credibility intervals for PFS and OS; significant results are emboldened. (TIF) [file pone.0283719.s009.tif]
